# University of Pennsylvania

**Published:** 1864-04

**Authors:** 


University of Pennsylvania.—The number of students attending the medical lectures during the past session was 401; and at the commencement held on the 12th of March the degree of M. D. was conferred on 101 candidates.

